# Digital health technologies in obesity: trajectory, emerging hotspots, and future perspectives

**DOI:** 10.3389/fmed.2026.1875769

**Published:** 2026-07-15

**Authors:** Qing Zhang, Jingjing Huang, Fangju Mao, Yingxia Wang, Wen Liu, Yangyao Peng

**Affiliations:** 1 Department of Integrated Health Service, Zhongnan Hospital of Wuhan University, Wuhan, Hubei, China; 2 Hubei International Science and Technology Cooperation Base for Research and Clinical Techniques for Brain Glioma Diagnosis and Treatment, Wuhan, Hubei, China; 3 Department of Plastic Surgery, Zhongnan Hospital of Wuhan University, Wuhan, Hubei, China; 4 Department of Joint Surgery, The First Affiliated Hospital of Yangtze University, Jingzhou, Hubei, China; 5 Department of Ophthalmology, Zhongnan Hospital of Wuhan University, Wuhan, Hubei, China; 6 Department of Cardiovascular Surgery, Zhongnan Hospital of Wuhan University, Wuhan, Hubei, China

**Keywords:** artificial intelligence, bibliometric analysis, digital health, obesity, weight management

## Abstract

**Background:**

Digital health technologies, including mobile applications, wearable devices, and artificial intelligence, have emerged as valuable platforms for addressing the global obesity epidemic. Despite a surge in academic output, the field lacks a macro-level comprehensive synthesis of its evolutionary logic, core research forces, and emerging frontiers.

**Methods:**

Literature published between 2016 and 2026 was retrieved from the Web of Science Core Collection and Scopus databases. Python was utilized for data cleaning, while descriptive statistical analyses were conducted using R., VOSviewer and CiteSpace were collaboratively employed to perform co-occurrence clustering, evaluate global collaboration networks, and conduct burst detection.

**Results:**

A total of 2,633 records were ultimately included. The field experienced a significant inflection point starting in 2021, with annual publication volume surging from 272 papers in 2021 to 564 papers in 2025. The United States (756 articles) and China (738 articles) are the primary contributors, with the United States acting as the central hub for global collaboration (centrality = 0.47). Tehran University of Medical Sciences is the most prolific institution (71 articles), whereas Harvard University occupies a critical bridging position (centrality = 0.18). Nutrients is the most active publishing venue (199 articles), and Obesity Reviews serves as a core foundational reference. High-frequency keywords are concentrated on physiological indicators and specific demographics, led by “adiposity” (110 occurrences), “adolescents” (102), and “blood pressure” (97). Keyword and reference burst analyses indicate a distinct shift in research focus: early research frequently cited macro-epidemiological data, whereas recent bursts from 2023 to 2026 highlight clinical translation, diagnostic test accuracy, and digital intervention evaluation.

**Conclusion:**

The bibliometric data suggest that academic focus on digital technology in obesity management has structurally shifted from its use as a passive observational tool to its application as an active clinical intervention strategy. To translate these trends into clinical efficacy, future advancements will likely depend on overcoming the digital divide across varying income regions, developing artificial intelligence-driven precision algorithms, and establishing synergistic cardiometabolic management models alongside novel pharmacotherapies.

## Introduction

Digital health refers to an emerging field that utilizes information technology, smart devices, and digital tools to elevate medical services and health management, and it is widely recognized as an innovative platform for accessing healthcare services, reducing medical costs, and strengthening public health. According to the foundational framework established by the World Health Organization (WHO), it integrates technologies such as electronic health records, telemedicine, wearable devices, artificial intelligence diagnostics, and health big data analytics (1). This field aims to optimize the allocation of medical resources, improve clinical efficiency, and empower individuals in disease prevention and health management. Digital health not only breaks the spatial and temporal constraints of traditional medicine to drive the development of personalized healthcare, but also reduces medical costs through data interconnectivity, serving as a core driver in the transition of the global healthcare system toward intelligence and precision.

Obesity is widely recognized globally as a complex chronic disease and one of the most critical public health challenges, rather than merely a “personal lifestyle failure.” Since 1975, the global prevalence of obesity has nearly tripled; by 2022, over 1 billion people were affected by obesity, accounting for approximately 13% of the global population. Recent projections indicate that by 2050, approximately 3.8 billion adults will be overweight or obese (2). Obesity significantly increases the risks of type 2 diabetes, cardiovascular disease, fatty liver disease, certain cancers, and premature mortality, serving as a primary driver of the global preventable disease burden (3, 4) Economically, obesity-related costs are projected to reach $4.32 trillion annually by 2035, accounting for approximately 3% of the global GDP (5).

Poor long-term adherence, weight regain, limited service accessibility, and high costs have restricted the overall efficacy of these interventions in curbing the obesity epidemic (6, 7). Even in high-income countries, implementation remains markedly inadequate, falling far short of covering all populations in need of treatment (8). The emergence of digital health technologies offers a breakthrough solution for the prevention and management of obesity. Researchers have advocated the utilization of online consultations, remote monitoring, and artificial intelligence to enhance service accessibility, individualization, and follow-up continuity, while simultaneously reducing costs to reach socioeconomically disadvantaged populations (7, 9, 10). Furthermore, leveraging big data analytics and machine learning models allows researchers to more accurately predict weight loss outcomes and identify high-risk populations. The integrated application of these technologies not only facilitates a paradigm shift from intermittent clinical encounters to continuous health monitoring but also establishes the technological foundation for personalized, precision interventions in obesity. Telemedicine and online platforms, which can increase consultation frequency, extend follow-up durations, and mitigate geographical and temporal barriers, are considered among the most pragmatic digital breakthroughs for the future of obesity care (11–13).

In recent years, academic output at the intersection of digital health and obesity has surged exponentially. While existing narrative and systematic reviews have rigorously evaluated the clinical efficacy of specific digital interventions, they are inherently limited to addressing narrow clinical questions across small, highly selected samples of literature. The fragmented nature of these micro-level findings makes it difficult for scholars to grasp the overall evolutionary logic and emerging trends of the field as a whole. As the volume of literature explodes, traditional review methods are inadequate for mapping the global research landscape from a macroscopic perspective. A critical knowledge gap remains regarding the structural relationships among core research forces, cross-disciplinary collaboration networks, and the dynamic shifting of research hotspots over time.

Therefore, a bibliometric analysis is uniquely appropriate and necessary to quantitatively and visually synthesize these massive datasets, overcoming the methodological constraints of manual systematic reviews. This study aims to conduct a visual bibliometric analysis of the literature in this field over the past decade to achieve three specific objectives: (1) to delineate the spatiotemporal publication trends and establish the global collaborative networks among countries and institutions; (2) to identify the core authors and foundational journals driving the field; and (3) to perform keyword and reference burst detection to reveal the evolutionary trajectory and predict future clinical frontiers. By fulfilling these objectives, we seek to provide a highly structured and macroscopic reference for policymakers, clinicians, and researchers to navigate the future of digital obesity care.

## Methods

### Search strategy and data sources

Data for this study were retrieved and downloaded from the Web of Science Core Collection (WoSCC) and Scopus on March 12, 2026, encompassing a search timeframe from January 1, 2016, to March 12, 2026. The overall research design and execution were conducted in strict alignment with the established methodological guidelines for bibliometric analysis proposed by Donthu et al. (14). Because obesity is the primary focus of this research, obesity-related terms were restricted to the title to yield more precise results.

The search strategy for WoSCC was as follows: (TS = (“digital health” OR “e-health” OR “m-health” OR “mobile health” OR “telemedicine” OR “telehealth” OR “artificial intelligence” OR “machine learning” OR “deep learning” OR “natural language processing” OR “mobile application*” OR “app” OR “apps” OR “website*” OR “web” OR “online” OR “software” OR “electronic health record*” OR “electronic medical record*” OR “smartphone*” OR “mobile phone*” OR “virtual reality” OR “augmented reality” OR “computer-aided diagnosis” OR “decision support system*” OR “expert system*” OR “internet” OR “5G” OR “personalized medicine” OR “information system*” OR “smart device*” OR “robot*”) AND TI = (“obesity” OR “obese” OR “overnutrition” OR “nutritional and metabolic diseases” OR “overweight” OR “fleshiness” OR “adiposity”)) AND LA = (English) AND DT = (Article OR Review) AND PY = (2016–2026). The search strategy for Scopus was as follows: (TITLE-ABS-KEY(“digital health” OR “e-health” OR “m-health” OR “mobile health” OR “telemedicine” OR “telehealth” OR “artificial intelligence” OR “machine learning” OR “deep learning” OR “natural language processing” OR “mobile application*” OR “app” OR “apps” OR “website*” OR “web” OR “online” OR “software” OR “electronic health record*” OR “electronic medical record*” OR “smartphone*” OR “mobile phone*” OR “virtual reality” OR “augmented reality” OR “computer-aided diagnosis” OR “decision support system*” OR “expert system*” OR “internet” OR “5G” OR “personalized medicine” OR “information system*” OR “smart device*” OR “robot*”) AND TITLE(“obesity” OR “obese” OR “overnutrition” OR “nutritional and metabolic diseases” OR “overweight” OR “fleshiness” OR “adiposity”)) AND (LIMIT-TO (DOCTYPE, “ar”) OR LIMIT-TO (DOCTYPE, “re”)) AND (LIMIT-TO (LANGUAGE, “English”)) AND PUBYEAR > 2015 AND PUBYEAR < 2027.

**Figure 1:**
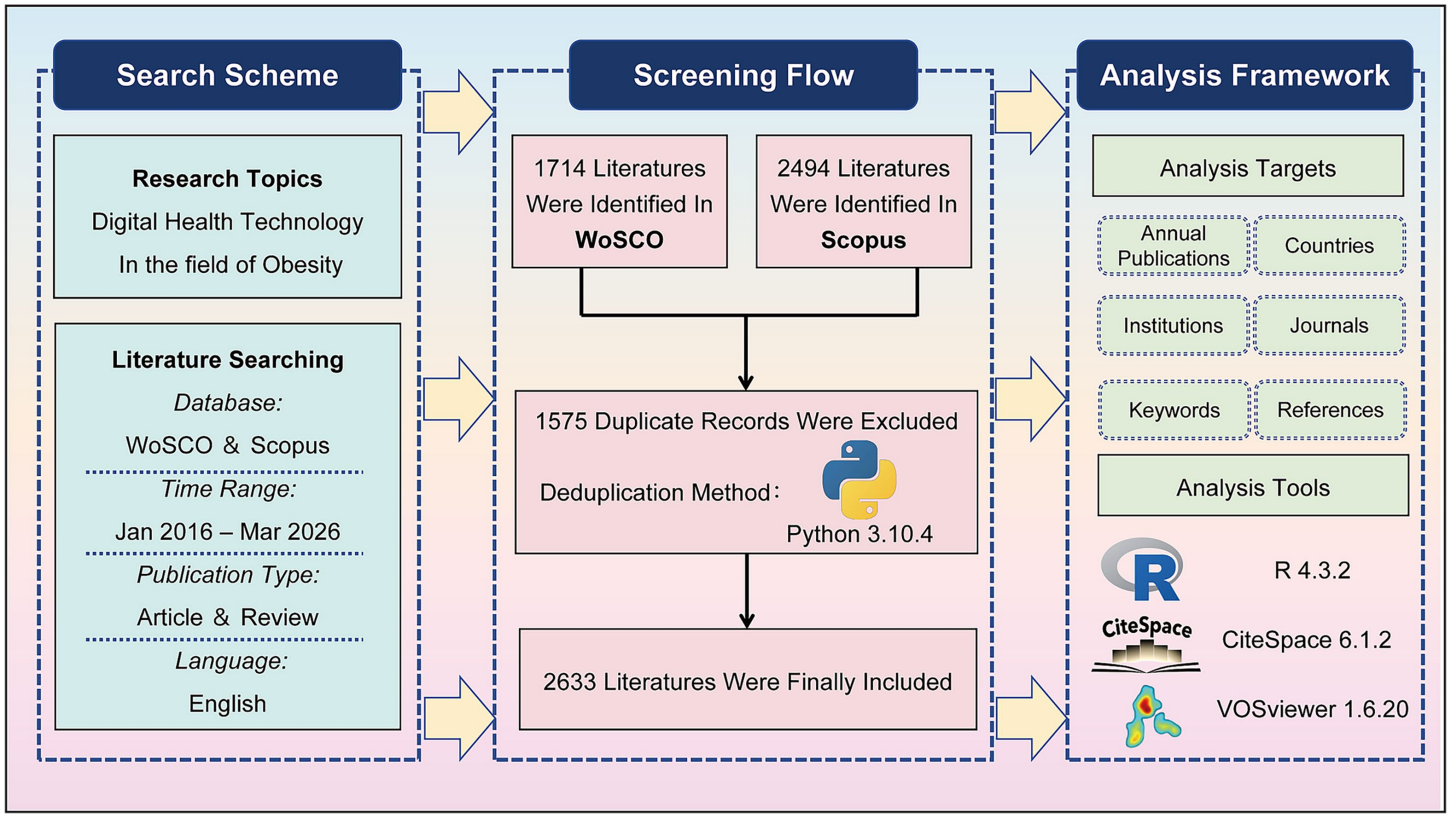
Methodological roadmap and literature selection process for the bibliometric analysis.

Given the preeminence of English in international academic discourse, only English-language publications were included to ensure the consistency and reliability of citation tracking and co-occurrence analysis. While this criterion may result in an underrepresentation of non-English academic literature, it ensured a high degree of homogeneity within the analytical sample. Eligible document types were restricted to articles and reviews. The literature inclusion and exclusion process, along with the technical roadmap of the study, are illustrated in Figure 1.

### Data analysis and visualization

Python (version 3.10.4) was utilized to clean, merge, and deduplicate the retrieved raw data. Subsequently, R (version 4.3.2) was employed for descriptive statistical analyses and to generate annual publication trend graphs and global geographical distribution maps, visually illustrating the spatiotemporal evolution of research output. The construction of knowledge graphs was collaboratively performed using VOSviewer (version 1.6.20) and CiteSpace (version 6.1.2). In VOSviewer, full counting was utilized, and association strength normalization was applied to construct co-occurrence and clustering networks for countries, institutions, journals, and keywords. The minimum co-occurrence thresholds were set dynamically, and the visualization was optimized by extracting the top 50 most active items. Regarding CiteSpace, the time-slicing was configured to 1 year per slice spanning 2016 to 2026. Node selection was based on the standard g-index (k = 25) threshold. To preserve the complete empirical network structure, no network pruning algorithms were applied, and node clustering was generated using the Log-Likelihood Ratio (LLR) algorithm. Concurrently, burst detection was performed using CiteSpace to identify research frontiers, executed with standard default parameters including a gamma value (*γ*) of 1.0 and a minimum duration of 1 year, multidimensionally revealing the knowledge structure and evolutionary hotspots of digital health in the field of obesity (Figure 1).

## Results

### Publication trends over time

**Figure 2:**
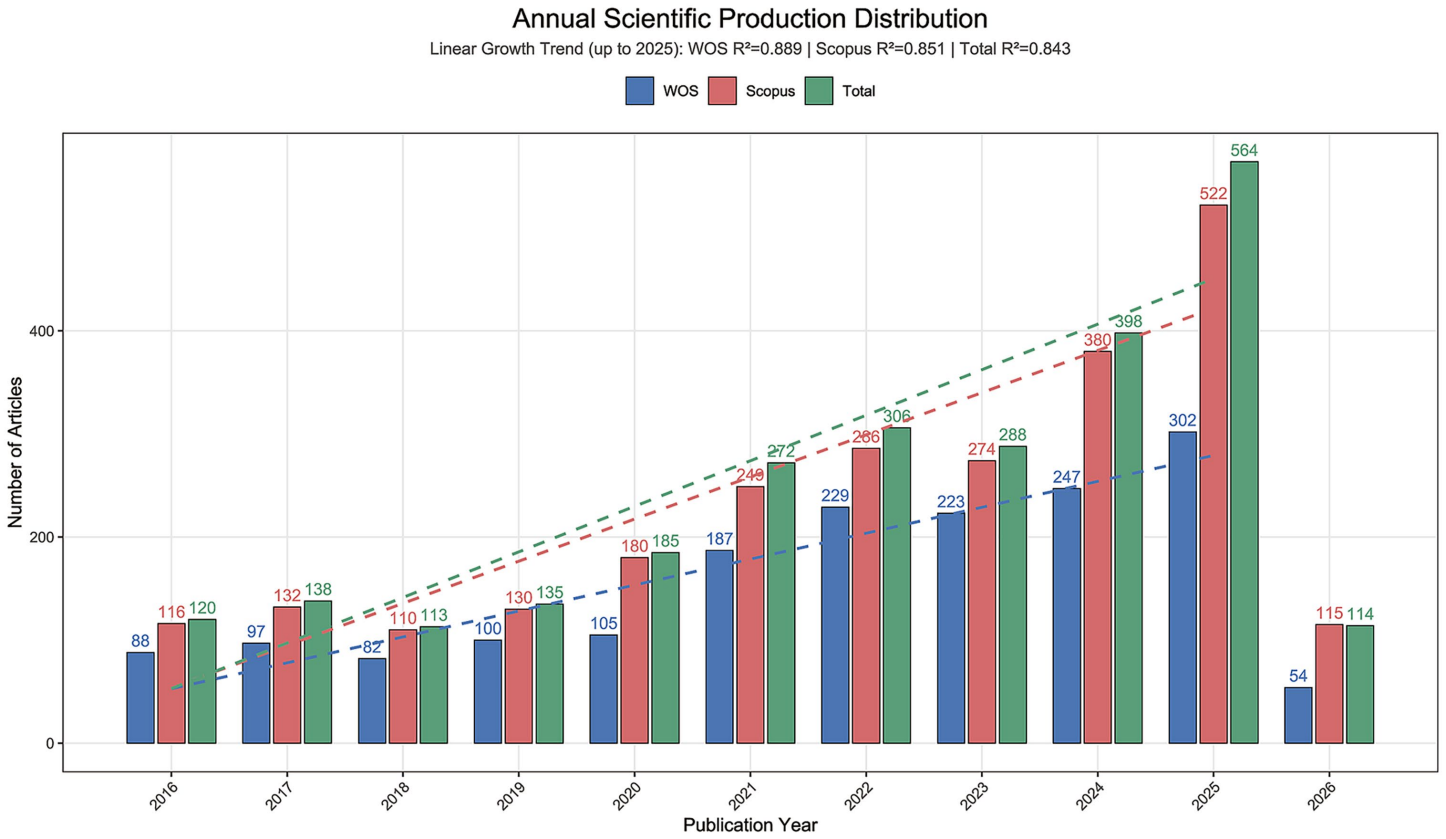
Annual scientific production distribution of digital health technologies in obesity research (2016–2026).

Between 2016 and 2020, research output regarding digital health technologies in the field of obesity remained in a phase of steady accumulation, with the total annual publication volume fluctuating slowly between 113 and 185 papers. Starting in 2021, the field experienced a critical inflection point characterized by exponential growth; the total publication volume surged from 272 papers in 2021 to 564 papers in 2025, essentially doubling the literature output. This robust upward trajectory demonstrated high consistency across two core databases, Web of Science and Scopus, and the linear growth trends through 2025 were statistically confirmed for both (Web of Science R2 = 0.889, Scopus R2 = 0.851). Furthermore, although the publication count for 2026 currently stands at 114, this only represents preliminary data collected up to March of that year; given the strong current research momentum and the highly fitted linear trends, the academic interest and publication volume in this field are projected to maintain a steady upward trajectory in the coming years (Figure 2).

### Most productive countries and institutions

**Figure 3:**
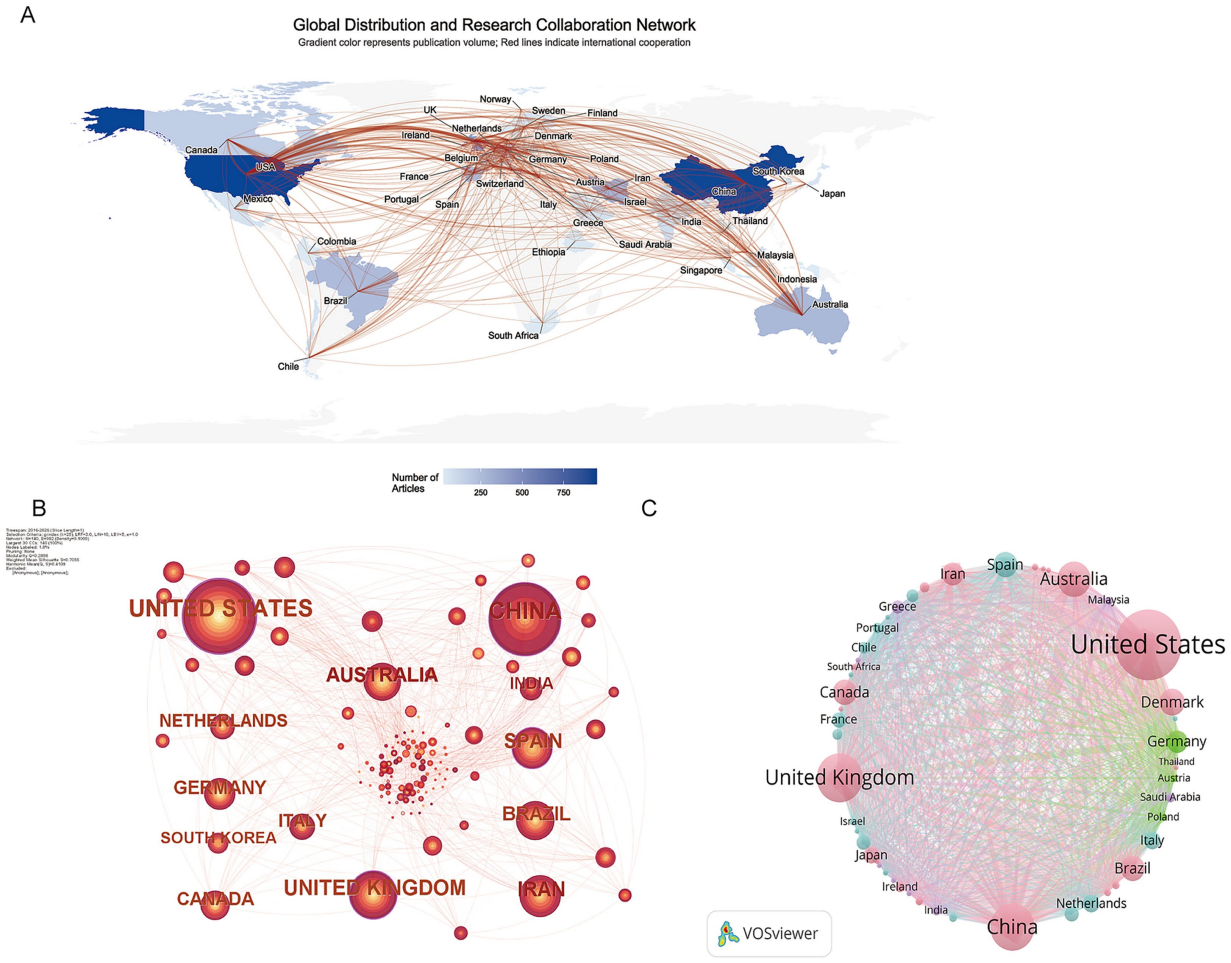
Global geographic distribution and country collaboration networks. **(A)** Global geographical distribution map illustrating publication volumes and international cooperation pathways (The blue color gradient corresponds to the total number of articles, while the red lines indicate collaborative links between countries) **(B)** The country collaboration network map generated by CiteSpace. **(C)** The country collaboration network visualization generated by VOSviewer.

Globally, research efforts at the intersection of digital health and obesity are predominantly concentrated in North America, East Asia, and Western Europe (Figure 3A). In terms of publication volume, the United States and China are the undisputed leaders, ranking first and second with 756 and 738 articles, respectively. The United Kingdom ranks third with 321 articles, followed by Iran (264 articles) and Spain (236 articles). Regarding academic influence, the United States maintains its dominant position with a total of 10,316 citations. The United Kingdom (5,841 citations) and China (5,469 citations) follow closely. Notably, although Denmark does not rank among the top ten in total publication volume, it leads in average citations per article (29.49), indicating the exceptional academic quality and widespread recognition of its research in this domain (Table 1).

**Table 1:** The publication status and citation status of country.

Rank	Country (Citespace)	Count	Centrality	Cite-country (Vosviewer)	Citations	Average Citation
1	United States	756	0.47	United States	10,316	10.8704
2	China	738	0.15	United Kingdom	5,841	13.6792
3	United Kingdom	321	0.23	China	5,469	5.8243
4	Iran	264	0.06	Australia	3,539	12.4175
5	Spain	236	0.13	Denmark	2,300	29.4872
6	Australia	226	0.07	Spain	2,111	8.311
7	Brazil	223	0.01	Canada	2,083	12.1105
8	Germany	154	0.07	Brazil	2,081	7.8528
9	Canada	142	0.04	Iran	1,757	5.7231
10	Italy	110	0.09	Germany	1,721	9.5083

The country collaboration network map generated via CiteSpace and VOSviewer (Figures 3B,C) clearly illustrates the tight academic connections between nations. The dense linkages in the global geographical distribution map (Figure 3A) further substantiate the frequency of intercontinental collaboration. Within this collaboration network, centrality serves as a crucial metric for evaluating a node’s significance as a structural bridge. The United States acts as the core hub of the global collaborative network, boasting the highest centrality (0.47) and playing an indispensable role in bridging academic exchanges across various countries and regions. The United Kingdom (centrality 0.23) and China (centrality 0.15) also constitute vital nodes for international cooperation. Overall, a multilateral collaborative paradigm centered around the United States, China, and the United Kingdom dominates the contemporary global research landscape of digital health in obesity.

In terms of institutional publication volume, Tehran University of Medical Sciences ranks first with an output of 71 articles, significantly outpacing other institutions. Harvard University and the Department of Clinical Nutrition rank second and third with 39 and 36 articles, respectively (Table 2). However, high productivity does not necessarily equate to high citation impact. Citation analysis via VOSviewer reveals that although the University of Pennsylvania does not rank among the top ten in publication volume, it amassed 1,650 total citations with a remarkable 50 citations per article, demonstrating its exceptional academic quality and core influence in the field of digital health and obesity. The University of Copenhagen and the University of Sydney follow closely with 887 and 870 total citations, respectively.

**Table 2:** The publication status and citation status of institution.

Rank	Institution (Citespace)	Count	Centrality	Cite- institution (Vosviewer)	Citations	Average citation
1	Tehran University of Medical Sciences	71	0.03	University Of Pennsylvania	1,650	50
2	Harvard University	39	0.18	University Of Copenhagen	887	26.8788
3	Department of Clinical Nutrition	36	0.15	Univ Sydney	870	28.0645
4	University of Sydney	35	0.08	Tehran University Of Medical Sciences	785	9.4578
5	National University of Singapore	30	0.06	Karolinska Institutet	730	28.0769
6	University of California System	29	0.08	Sichuan University	594	16.9714
7	Monash University	29	0.07	University Of Bristol	583	29.15
8	University of Copenhagen	28	0.17	Univ Granada	579	32.1667
9	Iran University of Medical Sciences	28	0.02	Harvard University	555	20.5556
10	Beijing Sport University	28	0.01	Deakin University	522	18.6429

**Figure 4:**
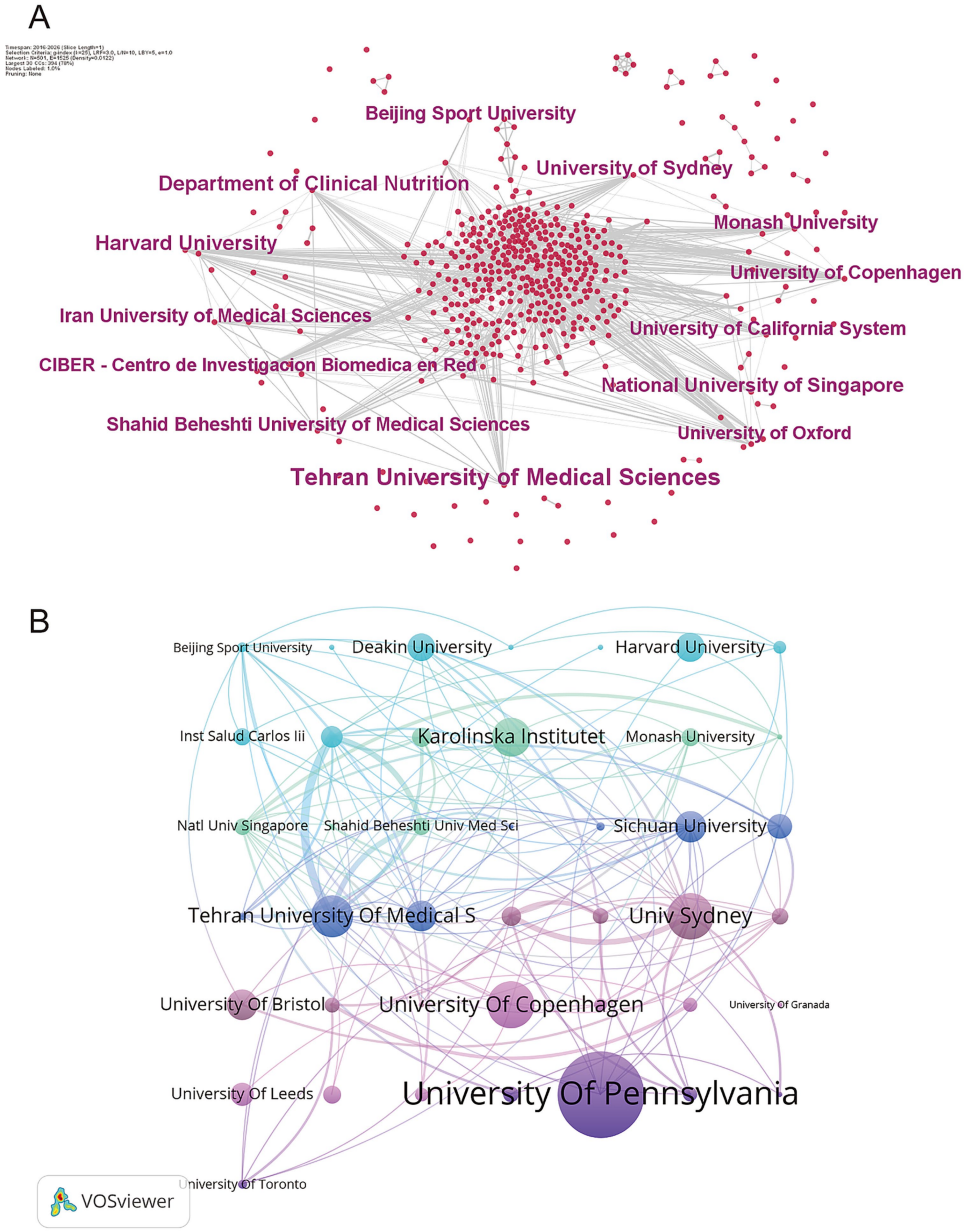
Institutional publication and collaboration network. **(A)** The institutional collaboration network map generated by CiteSpace. **(B)** The institutional co-occurrence and collaboration network visualization generated by VOSviewer.

The institutional co-occurrence map generated by CiteSpace and VOSviewer (Figure 4) reveals a collaborative network characterized by “multicenter clustering and local compactness.” Notably, an institution’s role as a collaborative bridge (i.e., centrality) does not necessarily align with its publication volume. Although Tehran University of Medical Sciences has the highest publication volume, its centrality is only 0.03 (Table 2), indicating that its research remains relatively independent or confined to specific collaborative circles. In contrast, Harvard University (centrality 0.18) and the University of Copenhagen (centrality 0.17) occupy the most critical hub positions within the map. Through extensive cross-regional collaboration, these two institutions serve as core nodes that effectively connect disparate global research teams, driving the intersection and integration of knowledge within the field.

### Influential journals and co-cited journals

**Figure 5:**
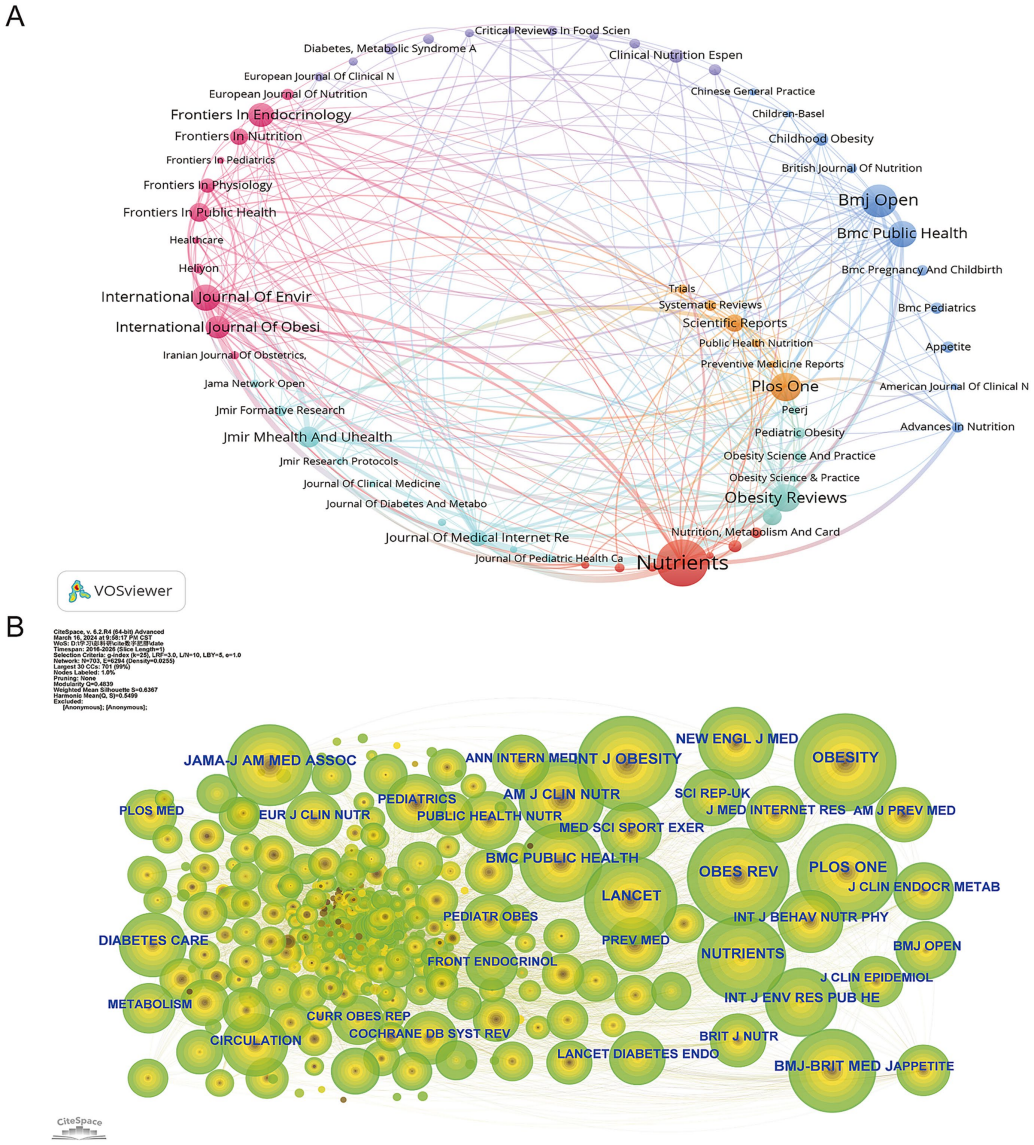
Active journals and co-cited journals network. **(A)** The network map of active publishing journals generated by VOSviewer. **(B)** The journal co-citation network visualization generated by CiteSpace.

The distribution of publications across journals intuitively reflects the disciplinary orientations and core dissemination channels of research in this field. As shown in Table 3, Nutrients is the most active publication in the field of digital health and obesity, ranking first with 199 articles (IF = 5.0); BMJ Open (112 articles, IF = 2.3) and PLOS One (88 articles, IF = 2.6) rank second and third, respectively. The journal network map generated by VOSviewer (Figure 5A) indicates that journals specializing in nutrition, public health, and comprehensive open-access medicine constitute the primary venues for publishing research outcomes in this domain.

**Table 3:** Top 10 active journals and co-cited journals analysis.

Rank	Cite-Journal (Citespace)	Count	Impact Factor	Journal (Vosviewer)	Documents	Impact factor
1	Obesity Reviews	842	7.4	Nutrients	199	5
2	Obesity	833	4.7	BMJ Open	112	2.3
3	Plos One	830	2.6	Plos One	88	2.6
4	International Journal Of Obesity	807	3.8	Obesity Reviews	86	7.4
5	Lancet	710	88.5	International Journal Of Environmental Research And Public Health	79	Removed
6	Nutrients	640	5	BMC Public Health	76	3.6
7	BMJ-British Medical Journal	618	43	Frontiers In Endocrinology	67	4.6
8	American Journal Of Clinical Nutrition	588	6.9	International Journal Of Obesity	61	3.8
9	JAMA-Journal Of The American Medical Association	584	55	JMIR Mhealth And Uhealth	55	6.2
10	BMC Public Health	549	3.6	Frontiers In Public Health	44	3.4

Journal co-citation frequency reflects a journal’s foundational support role in constructing the knowledge system of a field. CiteSpace analysis reveals that Obesity Reviews (842 citations, IF = 7.4), Obesity (833 citations, IF = 4.7), and PLOS One (830 citations, IF = 2.6) are the top three highly cited journals, constituting the core professional literature repository for this domain (Table 3; Figure 5B). Notably, world-leading general medical journals such as The Lancet (IF = 88.5), The BMJ (IF = 43.0), and JAMA (IF = 55.0) all rank among the top ten in the highly cited list (Table 3).

### Keywords co-occurrence and burst analysis

**Figure 6:**
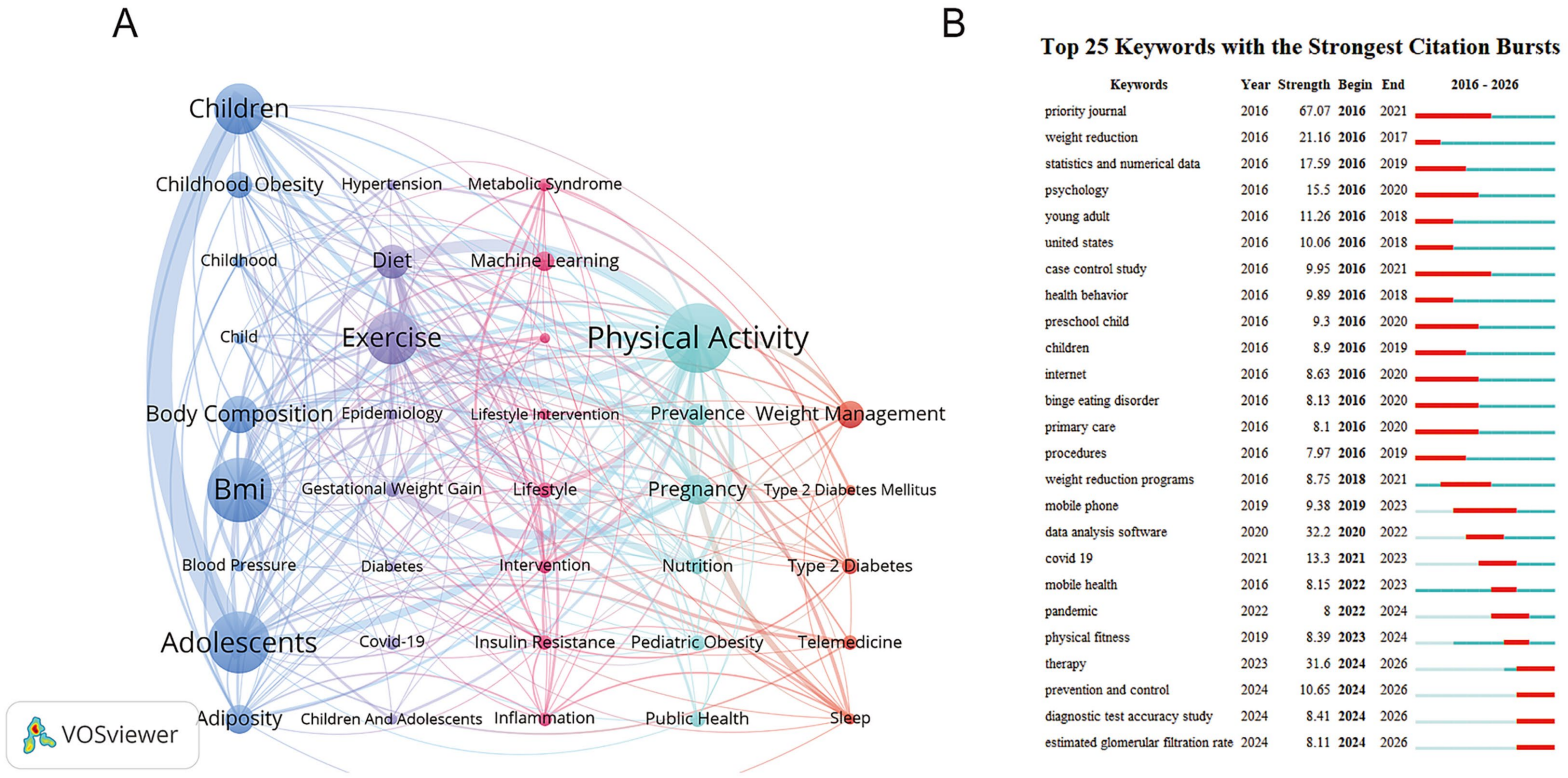
Keyword co-occurrence and thematic burst analysis. **(A)** The keyword co-occurrence network visualization generated by VOSviewer. The size of the nodes reflects the occurrence frequency of the keywords, and the connecting lines indicate their co-occurrence relationships. **(B)** The top 25 keywords with the strongest citation bursts generated by CiteSpace.

Co-occurrence analysis of keywords effectively reveals the core research themes within a specific domain. According to Table 4, the ten most frequent keywords in this field are adiposity with 110 occurrences, adolescents with 102, blood pressure with 97, BMI with 83, body composition with 80, child with 58, childhood with 52, childhood obesity with 46, children with 44, and children and adolescents with 41. These high-frequency terms are highly concentrated on specific demographic characteristics, namely children and adolescents, and core physiological indicators related to obesity, including adiposity, blood pressure, BMI, and body composition. The keyword co-occurrence network generated by VOSviewer, illustrated in Figure 6A, further demonstrates dense connections among these core nodes. They form the primary knowledge framework of current digital health research in obesity management, with major directions including children and adolescent populations, physical activity and exercise, and complication risk management like diabetes and hypertension.

**Table 4:** Top 20 high frequency keywords.

Rank	Keywords	Occurrences	Rank	Keywords	Occurrences
1	Adiposity	110	11	Covid-19	40
2	Adolescents	102	12	Diabetes	34
3	Blood Pressure	97	13	Diet	30
4	Bmi	83	14	Epidemiology	24
5	Body Composition	80	15	Exercise	24
6	Child	58	16	Gestational Weight Gain	23
7	Childhood	52	17	Hypertension	23
8	Childhood Obesity	46	18	Inflammation	23
9	Children	44	19	Insulin Resistance	22
10	Children And Adolescents	41	20	Intervention	22

Burst detection is utilized to track the dynamic shifts of academic focus over specific periods. The CiteSpace analysis presented in Figure 6B shows the top 25 keywords with the strongest burst strengths from 2016 to 2026. Chronologically, early research hotspots between 2016 and 2021 predominantly centered on weight reduction, psychology, and preliminary explorations of specific populations such as young adults and preschool children. Since 2019, alongside the proliferation of technologies, the research focus has shifted toward digital intervention modalities, with burst terms like mobile phone, mobile health, and data analysis software achieving dominance. In recent years from 2023 to 2026, the latest research frontiers in this field have evolved into prevention and control, diagnostic test accuracy study, and the monitoring of specific clinical indicators. Furthermore, the emergence of terms like COVID-19 and pandemic reflects the short-term yet profound driving impact of major public health emergencies on research directions within this domain.

### Citation analysis and highly cited references

**Figure 7:**
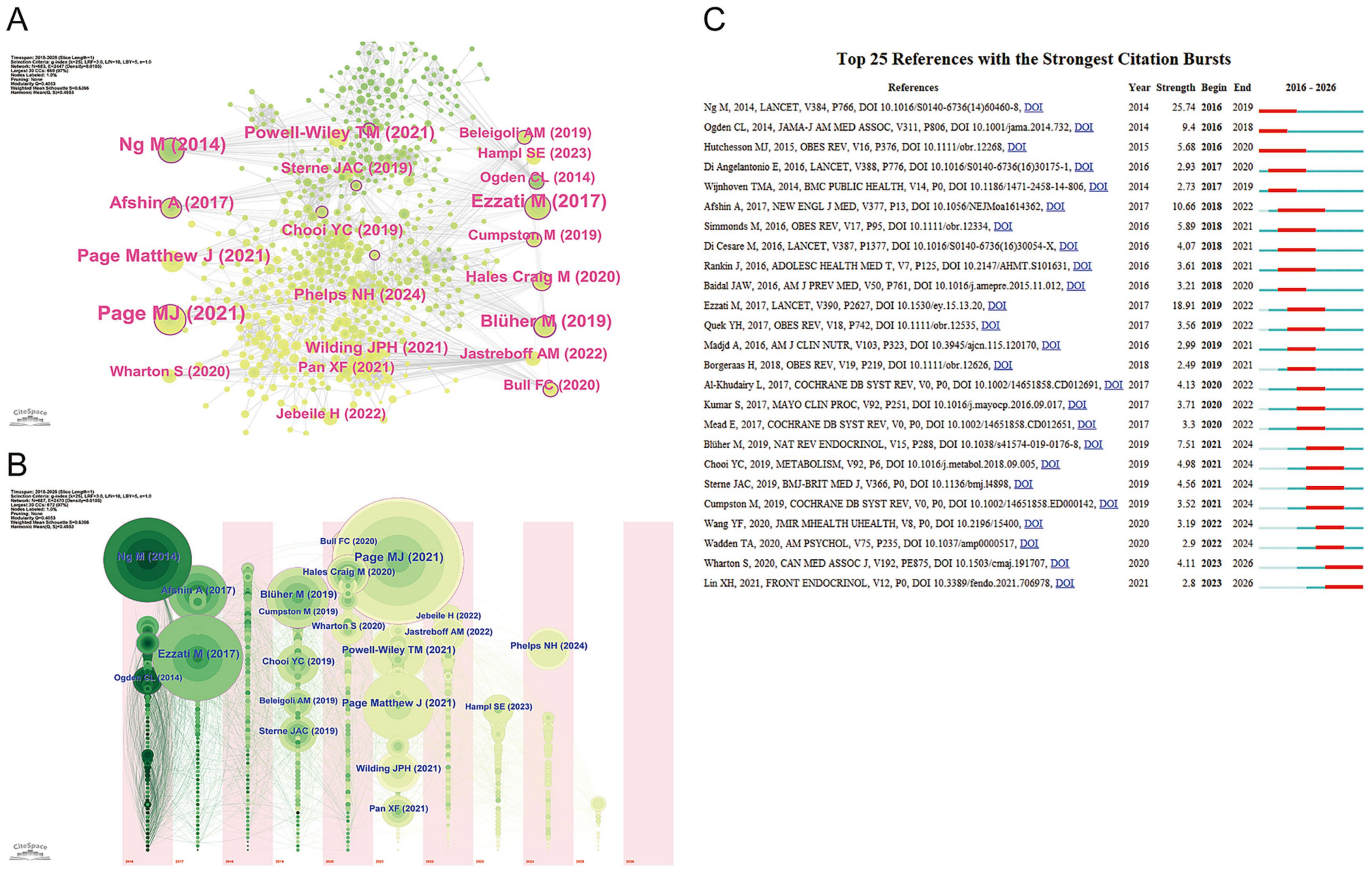
Reference co-citation network and burst analysis. **(A)** The reference co-citation network map generated by CiteSpace. The size of the nodes corresponds to the co-citation frequency of the references. **(B)** The timeline view of the reference co-citation network, illustrating the temporal evolution and cluster distribution of the highly cited references. **(C)** The top 25 references with the strongest citation bursts.

The top ten highly cited references in this domain are exclusively systematic reviews or clinical guidelines, underscoring the strong reliance of digital health and obesity research on high-level evidence-based medicine. Data from Table 5 indicate that the PRISMA 2020 statement and its explanation and elaboration document, published by Page et al. (15) in The BMJ, occupy the first and fourth positions on the co-citation list, respectively. This reflects the exceptionally high requirements for standardized reporting methodology when conducting systematic reviews in this field. Concurrently, macro-epidemiological surveys constitute the knowledge foundation of this research domain. Multiple Global Burden of Disease studies published in The Lancet and The New England Journal of Medicine rank at the top of the list, including the systematic analysis by Ng et al. on the global prevalence of overweight and obesity, and the pooled data by the NCD Risk Factor Collaboration on long-term trends in body-mass index. Beyond the methodological and epidemiological foundations, novel clinical interventions have also begun to attract immense attention. The review by Wilding on semaglutide treatment in adults, published in 2021 in The New England Journal of Medicine, entered the top ten, suggesting the formation of a critical cross-comparison benchmark between digital health management and frontier pharmacotherapies. The co-occurrence network in Figure 7 visually corroborates the tight knowledge transmission chains that have already formed among these core scholars.

**Table 5:** Top 10 highly cited references.

Rank	Title	Type	Count	Journal	Year
1	The PRISMA 2020 statement: an updated guideline for reporting systematic reviews (30)	Review	76	BMJ	2021
2	Worldwide trends in body-mass index, underweight, overweight, and obesity from 1975 to 2016: a pooled analysis of 2,416 population-based measurement studies in 128·9 million children, adolescents, and adults (31)	Review	52	Lancet	2017
3	Global, regional, and national prevalence of overweight and obesity in children and adults during 1980–2013: a systematic analysis for the Global Burden of Disease Study 2013 (32)	Review	51	Lancet	2014
4	PRISMA 2020 explanation and elaboration: updated guidance and exemplars for reporting systematic reviews (33)	Review	41	BMJ	2021
5	Obesity: global epidemiology and pathogenesis (5)	Review	36	Nature Reviews Endocrinology	2019
6	Obesity and Cardiovascular Disease: A Scientific Statement From the American Heart Association (34)	Review	34	Circulation	2021
7	Health Effects of Overweight and Obesity in 195 Countries over 25 Years (35)	Review	34	New England Journal of Medicine	2017
8	Worldwide trends in underweight and obesity from 1990 to 2022: a pooled analysis of 3,663 population-representative studies with 222 million children, adolescents, and adults (36)	Review	26	Lancet	2024
9	Once-Weekly Semaglutide in Adults with Overweight or Obesity (37)	Review	25	New England Journal of Medicine	2021
10	The epidemiology of obesity (16)	Review	24	Metabolism-clinical And Experimental	2019

Reference citation burst analysis can effectively capture the dynamic evolutionary trajectories of research frontiers within specific periods. The 25 references with the strongest burst strengths presented in Figure 7 clearly delineate three developmental stages in this field. In the early stage from 2016 to 2020, the research community relied heavily on basic epidemiological data; the burst literature during this period was overwhelmingly centered on the global obesity epidemiology report published by Ng in 2014. Starting in 2018, the academic focus began to diffuse toward the long-term health effects of obesity and specific population trends. Afshin’s study on the health effects of obesity across 195 countries and Ezzati’s landmark report on body-mass index trends in children and adolescents both exhibited strong citation bursts during this time. In the recent stage from 2021 to 2026, the bursting frontiers underwent a significant shift, pivoting comprehensively toward intervention evaluation, clinical guidelines, and digital technology applications. The concentrated bursts of three specific publications—Sterne’s risk of bias assessment tool published in The BMJ, Wharton’s clinical practice guidelines for adult obesity published in the Canadian Medical Association Journal, and Wang’s systematic review on the effectiveness of mobile health interventions published in JMIR mHealth and uHealth—indicate that the current research core has decisively transitioned from descriptive epidemiology toward the clinical translation, methodological standardization, and efficacy evaluation of digital interventions.

## Discussion

In this study, we comprehensively revealed the knowledge structure, research hotspots, and evolutionary trajectories of digital health technologies in the field of obesity by systematically analyzing literature from the Web of Science and Scopus databases spanning from 2016 to 2026. The results demonstrated a steady accumulation in early years followed by an explosive growth in annual publications since 2021, indicating that this field has rapidly transitioned from an initial exploratory phase into a period of widespread scholarly interest. Geographically and institutionally, the United States and China have emerged as the leading contributors, with top-tier institutions like Harvard University acting as crucial hubs driving global collaborative networks. Furthermore, high-impact journals such as Nutrients and Obesity Reviews serve as the core platforms for disseminating these scientific advances. Overall, the high accessibility and real-time monitoring capabilities of digital tools grant them unique advantages in long-term weight management and the prevention of cardiovascular complications. This trajectory aligns perfectly with the contemporary trends in modern public health and clinical medicine, which increasingly emphasize remote monitoring, personalized healthcare, and integrated chronic disease management.

### Drivers of exponential growth: technological evolution and public health crises

One of the most notable findings of this study is the exponential growth in publication volume concerning digital health in obesity since 2021. This trend is not coincidental but rather the result of the synergistic effects of technological maturation and the broader macroenvironment. First, smartphones, wearable devices, and mHealth applications achieved widespread proliferation during this period, providing accessible and cost-effective tools for continuous weight monitoring and lifestyle interventions (12). Second, the prominent bursts of the terms COVID-19 and pandemic in the keyword burst analysis underscore the catalytic role of major public health crises.

In recent years, research and practice regarding digital health in obesity have significantly accelerated, entering a fast track particularly following the onset of COVID-19. This has driven the expansion and normalization of remote weight management models (16, 17). Social distancing mandates and lockdown measures during the pandemic disrupted traditional in-person weight management clinics. Concurrently, obesity was established as an independent risk factor for severe COVID-19 infection, compelling healthcare systems and patients to pivot en masse toward telemedicine and digital interventions. This crisis-driven paradigm shift in healthcare delivery has provided unprecedented real-world data and application scenarios for the implementation of digital health technologies, cementing it as a persistent research hotspot (11).

### Transition of research foci: from epidemiology to precision clinical intervention

The evolutionary trajectories of reference co-citations and keywords reveal a distinct transition in research focus: scholarly interest is shifting from the use of digital technologies as passive observational tools to their evaluation as active interventional modalities. During the early research phase from 2016 to 2020, highly cited literature predominantly concentrated on Global Burden of Disease reports. In this period, digital technologies primarily facilitated the collection of large-scale epidemiological data. However, as research frontiers shift toward prevention and control and diagnostic test accuracy study, the academic focus has substantively advanced into the clinical translation phase.

The PRISMA 2020 statement emerged as the most frequently cited literature in this field, a phenomenon of landmark significance: it is by no means a mere methodological preference, but rather reflects that, following the proliferation of highly variable mHealth apps, this domain is undergoing a rigorous process of “separating the wheat from the chaff.” Researchers are urgently seeking to establish the real-world efficacy and evidence-based medical standing of novel digital therapeutics (DTx) through high-quality meta-analyses (12, 18, 19).

Crucially, the dense co-occurrence of the high-frequency terms “blood pressure” and “diabetes” signifies a fundamental deepening of the clinical endpoints in digital obesity management. This indicates that modern digital intervention strategies have transcended the singular goal of body-mass index reduction, instead comprehensively integrating metabolic syndrome indicators—such as blood pressure, glucose metabolism, and lipid profiles—into the core evaluation framework (20, 21). This progression toward synergistic cardiometabolic intervention aims to achieve the early interruption of cardiovascular event risk and comprehensive target organ protection through digitalized behavioral modification and precision monitoring. Indeed, although existing evidence indicates that absolute reductions in weight or body-mass index achieved via digital interventions are limited and predominantly short-term, these interventions demonstrate greater stability in improving waist circumference, body fat distribution, and several core metabolic parameters. This further corroborates the clinical rationale for transitioning from isolated weight loss to comprehensive cardiometabolic management (19). However, it is imperative to acknowledge that current studies are broadly constrained by substantial design heterogeneity, short follow-up durations, and inconsistent outcome measures. These limitations considerably weaken the evidence base for digital therapeutics as a standalone first-line treatment, highlighting an urgent need for long-term, standardized, large-scale randomized controlled trials.

### Mechanisms of digital interventions and adherence challenges during the weight-loss plateau

Transitioning from macroscopic epidemiological observation to individual clinical intervention requires an in-depth analysis of the underlying mechanisms by which digital technologies reshape behavior. The World Health Organization defines obesity as a chronic, relapsing disease requiring lifelong management, the core of which lies in maintaining a long-term negative energy balance. Compared to the lack of continuous supervision in traditional in-person clinics, digital health technologies—such as mobile applications and wearable devices—can provide immediate feedback and cognitive support in patients’ real-world daily settings through ecological momentary interventions. However, current evidence indicates that the weight loss effects of digital interventions are predominantly concentrated within the initial 3 to 6 months; thereafter, these effects gradually attenuate and are frequently accompanied by exceptionally high attrition rates, suggesting that long-term engagement has become the core bottleneck limiting the efficacy of digital therapeutics (22).

The underlying mechanism of this bottleneck lies in the exceptionally high execution burden and the inevitable physiological and psychological weight-loss plateaus. Once weight loss stalls, the motivation accumulated by patients during the initial honeymoon phase dissipates rapidly. Studies indicate that during the maintenance phase of behavioral weight loss programs, adherence to dietary and weight self-monitoring declines significantly over time; notably, once a patient’s recording days in a given month fall below 50%, the probability of subsequently resuming regular tracking drops below half, ultimately leading to comprehensive weight regain (23). To overcome this digital fatigue, the introduction of gamification, social comparison, and team collaboration mechanisms has been shown to exert potent anti-fatigue effects on physical activity and dietary behaviors in the short to medium term (24, 25). Intervention designs incorporating social comparison, team collaboration, and reward mechanisms can significantly elevate and prolong user engagement curves, partially mitigating the burnout induced by long-term self-monitoring. Nevertheless, how to deeply integrate these dynamic intelligent goals and virtual peer support strategies into real-world weight loss scenarios to achieve long-term weight maintenance benefits remains an urgent question, requiring validation through future real-world studies with larger sample sizes and extended follow-up periods.

### Global collaboration landscape and the challenge of the digital divide

In the collaborative network of countries and institutions, US and European institutions, such as Harvard University and the University of Copenhagen, exhibit high centrality and dominate the production of high-quality evidence-based medical research. Conversely, some high-output institutions, such as Tehran University of Medical Sciences, despite their significant publication volume, occupy relatively peripheral positions within the international network. This discrepancy reflects an imbalance in resource allocation and academic discourse within the current field. More importantly, the geographical distribution of publications reveals a significant lack of research participation from low- and middle-income countries (LMICs) (26). Considering that the global burden of obesity is rapidly shifting toward developing nations, this digital divide may exacerbate health inequalities (27). Future digital health research must strengthen transnational and cross-regional multicenter collaboration to develop digital intervention protocols that are more cost-effective, culturally adaptable, and less dependent on hardware, thereby reaching a broader range of vulnerable populations.

### Future perspectives: methodological challenges, AI integration, and synergistic management

Looking ahead, the application of digital health in the field of obesity will present two core clinical trends and one fundamental governance challenge. The first is the evolution toward artificial intelligence-driven precision interventions. Current mHealth efforts are largely limited to rule-driven “unidirectional outputs”; however, in the future, by leveraging deep learning algorithms, digital systems will be able to seamlessly integrate patients’ real-time physiological parameters, multi-omics data, and continuous behavioral trajectories to provide dynamically adjusted, personalized nutritional and exercise prescriptions.

The first clinical trend is the evolution toward artificial intelligence-driven precision interventions. Current mHealth efforts are largely limited to rule-driven “unidirectional outputs”; however, in the future, by leveraging deep learning algorithms, digital systems will be able to seamlessly integrate patients’ real-time physiological parameters, multi-omics data, and continuous behavioral trajectories to provide dynamically adjusted, personalized nutritional and exercise prescriptions. However, this evolution introduces critical ethical considerations regarding algorithmic bias. AI models trained predominantly on homogeneous datasets from specific socioeconomic or ethnic groups in high-income regions risk perpetuating clinical disparities. Ensuring diversity in AI training datasets and establishing transparent algorithms are fundamental prerequisites for the equitable deployment of these technologies.

The second trend is digital-drug synergy. As revealed by the list of highly cited references in this study, GLP-1 receptor agonists, represented by semaglutide, have emerged as a disruptive force in the field (28). However, while these novel weight-loss medications exhibit significant efficacy, they are generally fraught with clinical challenges, including rapid weight regain following drug cessation and the loss of lean body mass during follow-up. Consequently, digital health technologies will play an indispensable role as companion therapeutics in the future, utilizing digital tools to reinforce lifestyle modification during medication periods to achieve long-term weight maintenance and cardiovascular endpoint benefits.

Finally, the profound application of technology must be predicated on rigorous ethical considerations. As demonstrated by the keyword burst analysis, the increasing research reliance on mobile phones and data analysis software has led to significant privacy concerns regarding the collection of massive amounts of granular behavioral data (29). Given that obesity is often accompanied by deep-rooted social stigmatization, future big-data-based digital management models must strictly adhere to global data de-identification regulations. Precise interventions must be achieved under the premise of defending patient privacy rights and eliminating social stigma.

## Limitation

Although this study comprehensively delineates the knowledge graph of digital health technologies in the field of obesity, several objective limitations remain. First, the data retrieval was restricted to two mainstream databases—the Web of Science Core Collection and Scopus—and primarily focused on English-language literature; consequently, some excellent regional intervention studies indexed in other databases or non-English journals may have been omitted. Second, when performing natural language processing and data cleaning, bibliometric software inevitably introduces minor extraction biases, such as imprecise normalization of individual author homonyms or affiliated institutional names. Finally, metrics such as citation frequency are subject to an inherent time-lag effect. Highly groundbreaking research published in recent years may not yet have fully manifested its true academic impact within core citation lists or burst analyses due to the short duration for accumulating citations. Furthermore, the incomplete data for 2026 are insufficient to comprehensively reflect the definitive research output trends of the current year. Lastly, it must be emphasized that as a bibliometric analysis, this study maps macroscopic research trends based on publication metadata; therefore, it cannot be used to draw definitive conclusions regarding the actual clinical effectiveness of specific interventions, which must be systematically assessed through rigorous clinical trials.

## Conclusion

This study, through multidimensional bibliometric and visualization analyses, systematically delineates the evolutionary trajectory and knowledge framework of digital health technologies in the field of obesity management between 2016 and 2026. The findings indicate that after a phase of steady early accumulation, academic output in this field experienced exponential growth starting in 2021, reflecting the macroscopic global healthcare trend toward remote and digitalized chronic disease management. The United States and China remain the core dominant forces in both scientific productivity and international collaboration. The future of the field lies in three strategic pillars: the integration of AI-driven precision algorithms, the exploration of digital-drug synergy with frontier GLP-1 therapies, and the expansion of multicenter international collaborations. These advancements are essential to achieving sustainable, equitable, and evidence-based global obesity control.
